# A Dynamic, D-dimer-based Thromboprophylaxis Strategy in Patients with COVID-19

**DOI:** 10.12688/f1000research.146710.3

**Published:** 2024-11-11

**Authors:** Lantarima Bhoopat, Anastasia Martynova, April Choi, Pattharawin Pattharanitima, Semi Han, Senxi Du, Ibrahim Syed, Catherine Chan, Esther E Oh, Zea Borok, Janice Liebler, Melissa Lee Wilson, Pichaya Tantiyavarong, Casey O Connell

**Affiliations:** 1 Department of Medicine, Keck School of Medicine, University of Southern California, Los Angeles, California, 90089, USA; 2 Department of Internal Medicine, Thammasat University, Pathum Thani, Pathum Thani, 12120, Thailand; 3 Division of Hematology and Oncology, University of California Irvine, Orange, California, 92868, USA; 4 Los Angeles County + University of Southern California Medical Center, Los Angeles, California, 90089, USA; 5 Department of Medicine, University of California San Diego, San Diego, California, 92037, USA; 6 Department of Population and Public Health Sciences & SC-CTSI, University of Southern California, Los Angeles, California, 90089, USA

**Keywords:** D-dimer, Thromboprophylaxis, COVID-19

## Abstract

**Background:**

COVID-19 pandemics increases venous thromboembolism (VTE) risk during hospitalization, despite prophylactic anticoagulation. Limited radiological diagnosis in pandemic requires a guided protocol for anticoagulant adjustment.

**Methods:**

This retrospective cohort study was conducted at a single center as part of a quality improvement program evaluating the efficacy and safety of anticoagulation protocols. The study focused on implementing a guideline for anticoagulant dosing protocol based on dynamic changes in D-dimer levels in COVID-19 hospitalized patients. The dosing guideline allowed for dose escalation from standard prophylactic levels to escalated prophylactic or therapeutic levels, depending on the patient's risk profile for VTE. The primary endpoints included in-hospital survival comparing between fix and dynamic adjustment treatment groups. Secondary endpoints encompassed major and clinically relevant non-major bleeding (CRNMB) events, incidence of breakthrough thrombosis, length of hospitalization and ICU stay, days of mechanical ventilator use, and survival duration.

**Findings:**

Among the 260 COVID-19-infected patients hospitalized between March 15th and June 15th, 2020. The patients received fixed anticoagulant dosage in 188, 72.3%) patients, while 72 (27.7%) were up-titrated according to the protocol. In-hospital survival at 30 days demonstrated superiority among patients whose anticoagulation was up-titrated to either escalated prophylactic or therapeutic (80.2%) compared to receiving fixed anticoagulant dosage (51.3%) (p=0.01). Bleeding events were significantly higher in up-titrate group (12.5%) compared to fixed anticoagulant dosage group (2.13%). Most of them are CRNMB.

**Conclusion:**

A dynamic, D-dimer-based dose escalation of anticoagulation for hospitalized patients with COVID-19 holds promise in improving in-hospital mortality rates without a significant increase in fatal bleeding events.

## Introduction

Severe acute respiratory syndrome coronavirus 2 (SARS-CoV-2, COVID) has resulted in significant morbidity and mortality worldwide. One important mechanism for its adverse impact appears to be its promotion of arterial and venous thrombosis, endothelial dysfunction and enhanced angiogenesis, particularly in the lungs. ^1^ Early data from China found an association between D-dimer and mortality, but the actual incidence of thrombotic events was not reported. ^2^ A subsequent study showed that heparin or low molecular weight heparin (LMWH) improved survival in patients with sepsis-induced coagulopathy (SIC) score ≥ 4 or D-dimer value greater than 6 times the upper limit of normal (ULN). ^3^ However, despite standard heparin or LMWH thromboprophylaxis, the cumulative incidence of thrombotic events in COVID-infected patients stay high almost 50% over critically ill patients in an intensive care unit (ICU) in the Netherlands. ^4^^,^^5^ While the recent guidelines support the use of standard thromboprophylaxis in critically ill COVID-19 patients over therapeutic dose anticoagulant in term of survival benefit and days of organ support. However, the use of therapeutic dose heparin in noncritically ill COVID-19 patients reduce the need for mechanical ventilation and improve survival. ^6^

The concern for breakthrough thrombosis in hospitalized patients with limitations on radiologic testing access, prompted interest in using laboratory-based guide to anticoagulant dose adjustment at our large metropolitan public hospital. A large retrospective cohort shows a cut-off d-dimer of 1.8 mcg/ml FEU with 70% accuracy in diagnosis of acute symptomatic pulmonary embolism (PE) patients that are associated with worse mortality and morbidity outcomes. ^7^ A large retrospective study proposes the D-dimer cut-off of 2495 ng/ml (4.9 mcg/ml FEU) for diagnosis of asymptomatic PE in COVID-19 patients. ^8^ A systematic review also shows an association between D-dimer levels between 1000-4800 microgram/L (2-9.6 mcg/ml FEU) and pulmonary embolism in patients with COVID-19. ^9^

Based on COVID-related limitation on access to radiologic testing for deep vein thrombosis (DVT) and pulmonary embolism (PE) early in the epidemic, we implemented a D-dimer-stratified anticoagulation protocol for COVID patients as part of a quality improvement protocol for hospitalized patients who need anticoagulant. This protocol is dynamic and adjusted in associated with clinical of the patients and d-dimer level. The primary endpoint was in-hospital survival of who received anticoagulant titration according to the protocol compared with those who received fix anticoagulant dose since the beginning. Secondary endpoints included safety outcome, incidence of breakthrough thrombosis, length of hospitalization and ICU stay, days of mechanical ventilator use, and survival duration.

## Methods

**Figure 1.**
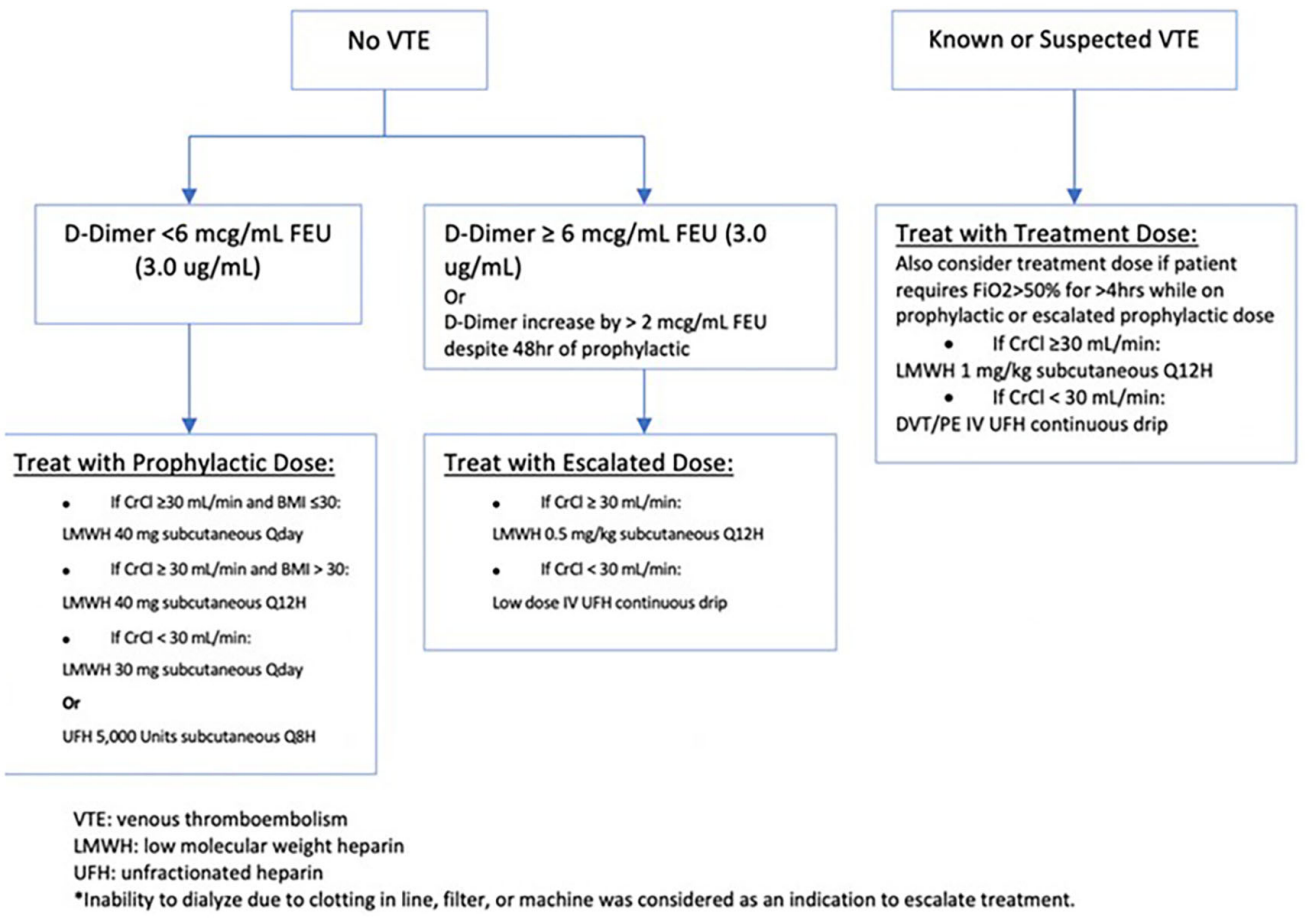
Anticoagulation Treatment Protocol Based on D-Dimer levels.

This single-center retrospective cohort study is part of an ongoing quality improvement initiative evaluating the use of an anti-factor Xa-driven heparin protocol, which includes a low-dose intravenous (IV) unfractionated heparin (UFH) option. A guideline for the use of anticoagulation in COVID-infected patients was implemented in February 2020 to standardize the management of these patients ( Figure 1). The inclusion criteria include all patients admitted with COVID-19 pneumonia confirmed by PCR testing between March 15th and June 15th, 2020. Patients who required therapeutic anticoagulation for other indications at the time of admission, those who presented with radiographically confirmed DVT or PE and those who died within 24 hours of anticoagulant treatment were excluded from this analysis. Baseline D-dimer level was obtained on admission and every 24-48 hours during hospitalization. Levels of D-dimer were measured by means of the STA® - Liatest® D-Di Plus assay (Roche Diagnostics SpA, Milan), an immuno-turbidimetric assay for the quantitative determination of D-dimer in venous plasma. Those who presented with radiographically confirmed DVT or PE were started on treatment dose anticoagulation as per the hospital protocol and were therefore excluded from this analysis. Those without existing thrombosis were stratified into two groups based on their D-dimer: those with a D-dimer below 6 mcg/ml FEU (3.0 ug/mL) were given standard prophylactic anticoagulation and those with a D-dimer above this cutoff received escalated prophylactic dosing (LMWH 0.5 mg/kg subcutaneous every 12 hours or low dose heparin intravenous continuous drip titrated to an anti-Xa level of 0.1-0.3). Subsequent dosing increases were made based on a rise in D-dimer to above 6 mcg/mL FEU, an increase of more than 2 mcg/mL FEU/mL despite >48 hours of prophylactic anticoagulation or inability to dialyze due to clotting in line, filter, or machine. Patients who demonstrated acute respiratory decompensation while receiving prophylactic or escalated prophylactic dosing had further escalation to full therapeutic dosing.

Based on our hospital’s anticoagulation protocol ( Figure 1), we categorized anticoagulant use as follows:

*Standard Prophylactic* which includes subcutaneous UFH 5,000 units every 8 hours (if creatinine clearance <30 mL/min), or subcutaneous LMWH with enoxaparin 40 mg once daily (40 mg twice daily if BMI>30; 30 mg once daily if Creatinine Clearance <30 mL/min)

*Escalated Prophylactic* which includes low-dose IV UFH titrated to achieve an anti-factor Xa level of 0.1-0.3 anti-Xa units (if creatinine clearance <30 mL/min) or enoxaparin 0.5 mg per kilogram body weight subcutaneously every 12 hours (if creatinine clearance ≥ 30 mL/min)

*Standard Therapeutic* dose used, which includes therapeutic dose IV UFH titrated to achieve an anti-factor Xa level of 0.3-0.7 anti-Xa units, or enoxaparin 1 mg per kilogram body weight every 12 hours during the hospitalization.

Patients treated with different anticoagulation regimens during their hospitalization were categorized into 2 categories based on their requirement of dosing up-titration as who received up-titrating according to the protocol and who received fixed anticoagulant dosing ( Figure 1).

We collected baseline demographic data including age, sex, body mass index (BMI), ethnicity, comorbidities, baseline inflammatory markers, and clinical parameters associated with disease severity including intensive care unit (ICU) admission, requirement for high flow nasal cannula (HFNC), mechanical ventilation (MV), use of vasopressor for maintenance of hemodynamic stability, and sepsis-induced coagulopathy score (SICS) ^10^ and also COVID-specific therapies.

The primary endpoints assessed were 30 day-in-hospital survival based on patients who were titrated to either escalated or therapeutic dose compared to fixed dose anticoagulant. We also adjust the potential covariates factors that affect in-hospital survival, such as D-dimer levels, age, ethnicity, BMI, comorbidities, ICU admission, mechanical ventilation, HFNC, SIC score, COVID treatment, thrombosis, and bleeding events. Secondary end points included bleeding outcome according to International Society on Thrombosis and Hemostasis (ISTH) defined major and clinically relevant non-major bleeding events. ^11^ Breakthrough thrombosis was recorded if identified on a radiologic study (venous duplex ultrasonography, CT-pulmonary angiogram, CT with contrast, CT or MRI brain) during the course of the hospital stay. Duration of hospitalization and ICU stay, and days of mechanical ventilator use.

### Statistical analysis

To compare demographic data among treatment groups, ANOVA or Kruskal-Wallis were used for continuous data, and Pearson’s Chi-Squared or Fisher’s exact test for categorical data. The bleeding outcomes among the groups were compared using exact probability tests without p-value adjustment for multiplicity. Dichotomous secondary outcomes were assessed using univariate logistic regression. In-hospital survival stratified by treatment groups was tested using Log rank testing and visualized using Kaplan Meier estimates. The Cox proportional hazard model was used to identify potential predictive factors for in-hospital mortality. We evaluated the following potential covariates: D-dimer levels, age, ethnicity, BMI, comorbidities, ICU admission, mechanical ventilation, HFNC, SIC score, COVID treatment, thrombosis, and bleeding events. Variables were included in the model if they were either predictors or confounders, defined as any variable that alters the effect size by >15% when included in the model. Scale Schoenfeld residuals were tested in the final model and showed a valid proportional hazard assumption. Additionally, we compared and graphically presented the mean profiles over time of D-dimer among treatment groups by random-intercept linear mixed model. This model incorporated the correlation of D-dimer measures within individuals. The fixed effects were time measuring D-dimer, treatment groups, and interaction of both variables. Restricted cubic spline with 3 degrees of freedom was applied for time to relax linearity assumption. All analyses were performed by STATA SE 17.0(Stata Corp, College Station, TX). We present 95% confidence interval of any effect measures with two-sided p value of 0.05 for statistical significance. This study is reported in accordance with the Strengthening the Reporting of Observational Studies in Epidemiology statement for observational studies.

According to COVID infection was a new emerging disease at the time of the study conducted, all eligible patients meeting inclusion criteria were included in the study. However, 2 patients had missing data on the primary outcome and were subsequently excluded from the analysis. Power was calculated retrospectively using STATA SE 17.0 revealing power of 0.99 at an alpha error (significant) threshold of 0.05STATA SE 17.0 used for all statistical analysis is licensed to the Faculty of Medicine, Thammasat University. RStudio, An open-access alternative software capable of performing equivalent functions for statistical analysis, is available for download at https://posit.co/download/rstudio-desktop/.

Formulae

The syntax for calculating power for two proportions in Stata is:

powertwoproportions p1 p2,alpha(level)power(power)n1(#)n2(#)testtype(type)

p1 and p2: The proportions of the two groups you are comparing.

alpha: The significance level

power: The desired power of the test

n1 and n2: The sample sizes for the two groups.

testtype: The type of test to conduct (e.g., two-sided or one-sided).

. power twoproportions 0.802 0.513, test (chi2) n1(72) n2(188)

Estimated power for a two-sample proportions test

Pearson's chi-squared test

H0: p2 = p1 versus Ha: p2 != p1

Study parameters: alpha = 0.0500

N = 260

N1 = 72

N2 = 188

N2/N1 = 2.6111

delta = -0.2890 (difference)

p1 = 0.8020

p2 = 0.5130

Estimated power: power = 0.9956

## Results

### Demographic data

The cohort comprised 260 hospitalized COVID-infected patients, with 137 of them being male (72.9%). The initial anticoagulant dosage was categorized as prophylactic for 242 patients (93.1%), escalated prophylactic for 6 patients (2.3%), and therapeutic for 12 patients (4.6%). Most patients (188, 72.3%) received a fixed anticoagulant dosage throughout their hospital stay. Among those receiving fixed anticoagulant dosing, they were further classified into fixed prophylactic dosing (171, 90.9%), fixed escalated prophylactic dosing (6, 3.2%), and fixed therapeutic dosing (11, 5.85%) treatment. There were 72 (27.7%) patients who had their anticoagulant doses adjusted to either escalated or therapeutic doses over the course of hospital stay based on their D-dimer trend ( Table 1). The patients in the anticoagulant adjustment group were significantly older than the patients in the fixed prophylactic group (mean age 60.7 ± 12.2 vs 54.6 ± 15.9 years, p<0.01). Most of the patients (178, 68.4%) had comorbidities that were associated with higher thrombotic risk. The most common thrombotic risk is cardiovascular risk factors.

**Table 1.** Demographic and Clinical Characteristics of the Study Population According to Heparin Adjustment Protocol.

		Fixed dose anticoagulant		Dynamic up-titrate anticoagulant		
Variable	Total N	N	Mean ± SD or median (min, max) or Count (%) ^a^	N	Mean ± SD or Count (%) ^a^	p-value ^b^
Age, years	260	188	54.6 ± 15.9	72	60.7 ± 12.2	<0.01
BMI	254	182	28.9 ± 7.2	72	31.1 ± 8.8	0.04
**Sex**	260	188		72		0.12
Female			51 (27.1)		13 (18.1)	
Male			137 (72.9)		59 (81.9)	
**Race/Ethnicity**	260	188		72		0.43
Native American			1 (0.5)		0 (0.0)	
Asian			8 (4.3)		1 (1.3)	
African American			4 (2.1)		5 (6.9)	
White (non-Hispanic)			13 (6.9)		4 (5.5)	
Hispanic			140 (74.5)		54 (75.0)	
Unknown/not reported			22 (11.7)		8 (11.1)	
**Comorbidity**	178	123		55		0.08
CV Risk Factors			106 (86.2)		46 (83.6)	0.65
Previous CAD			16(8.5)		7 (9.7)	0.76
Previous VTE			4 (3.2)		0 (0)	0.32
Cancer			13 (10.6)		5 (9.1)	1.00
Atrial Fibrillation			5 (4.1)		6 (10.9)	0.10
HIV			1 (0.8)		3 (5.4)	0.08
Autoimmune Disease			2 (1.6)		0 (0)	1.00
Pulmonary Disease			16 (13.1)		8 (14.5)	0.80
Chronic kidney disease			34 (27.6)		24 (43.6)	0.03
**Treatment**	260	188		72		
Remdesivir			4 (2.1)		7 (9.7)	0.01
Hydroxychloroquine			11 (5.8)		14 (19.4)	<0.01
Azithromycin			97 (51.6)		55 (76.4)	<0.01
Anti-IL-6			0 (0)		1 (1.4)	0.27
Convalescent plasma			11 (5.8)		11 (15.3)	0.01
Steroid			38 (20.2)		41 (56.9)	<0.01
**Severity**	260	188		72		
ICU admission			69 (36.7)		62 (86.1)	<0.01
HFNC requirement			54 (28.7)		50 (69.4)	<0.01
Mechanical Ventilation			25 (13.3)		48 (66.7)	<0.01
Vasopressor			21 (11.2)		38 (53.5)	<0.01
**Laboratory**						
Hemoglobin (g/dL)	260	188	13.4 ± 1.9	72	13.6 ± 2.3	0.58
Platelets (cu.mm.)	255	184	254,720 ± 120922	71	217,126 ± 106915	0.02
**Coagulogram**						
PT (sec)	184	124	14.2 ± 1.6	60	14.9 ± 1.6	0.01
aPTT (sec)	68	45	33.8 ± 5.2	23	32.8 ± 4.3	0.43
**Inflammatory marker**						
D-dimer (mcg/mL FEU)	219	149	1.0 (0.2, 20)	70	1.6 (0.2, 20)	<0.01
Peak D-dimer (mcg/mL FEU)	216	146	1.2 (0.2,20)	70	7.5 (0.6,20)	<0.01
LDH (U/L)	178	122	400.9 ± 180.9	56	480.3 ± 170.9	<0.01
CRP (mg/L)	211	149	139.7 (0.8, 368.5)	62	170.5 (6.6, 402.9)	0.16
Ferritin (ng/mL)	185	126	849.5 (6, 8871)	59	785 (85, 13170)	0.84
SIC Score	175	118	1.94 ± 1.1	57	2.6 ± 1.1	<0.01

^a^ Continuous variables are presented as means and standard deviations (SD) or median with minimum and maximum, while categorical variables are presented as counts and percentages.

^b^ P-values were obtained using one-way ANOVA for continuous variables and Pearson’s Chi-Squared or Fisher’s Exact Test for categorical variables, as appropriate.

^*^ Statistically significant difference compared to prophylactic at p<0.05 with Bonferroni p-value correction (ANOVA).

The patients who were treated with anticoagulant adjustment received more aggressive treatment for COVID infection such as remdesivir, azithromycin, hydroxychloroquine, convalescent plasma, and steroid according to their severe clinical course, while 40.4% of the patients who received fixed anticoagulant group received only supportive treatment. The severity of patients in anticoagulant adjustment groups were referred to a higher rates of ICU admission (86.1%), higher rate of high flow nasal cannula (HFNC) requirement (69.4%), higher rate of mechanical ventilator (MV) requirement (66.7%) and higher rate of vasopressor use (53.5%) compared to fixed anticoagulant group. The sepsis induced coagulopathy scores (SICS) were also significantly higher in this group (53.5%), correlated with higher lactate dehydrogenase (LDH), d-dimer level at baseline and at peak amount during admission as shown in Table 1.

### Survival outcome

**Figure 2.**
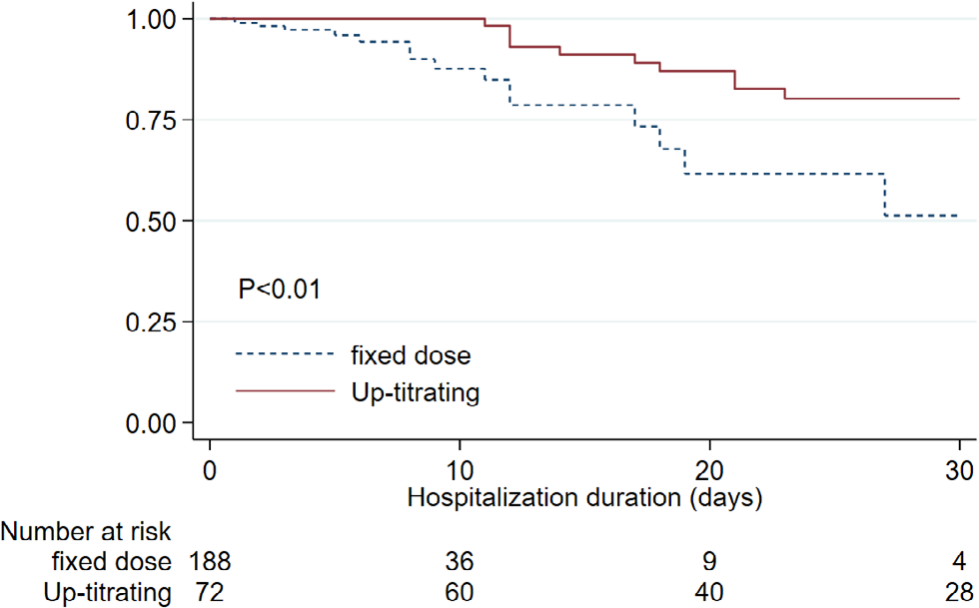
Kaplan-Meier estimates for in-hospital survival of COVID-infected patients comparing up-titrated anticoagulant group to those who received fixed anticoagulant dose since the beginning.

The mortality rate in this cohort of hospitalized COVID-infected patients was 12.7%. When we compared 30 days in-hospital survival of the patients who received anticoagulant adjustment (up-titrating) according to the protocol to whom that received fixed anticoagulant dosing, the up-titrated group showed statistically significantly higher in-hospital survival until discharge at 30 days of 80.2% compared to 51.3 % in the fixed anticoagulant group ( Figure 2).

Based on the multivariable Cox regression, we found factors that independently increased in-hospital mortality that include increasing age (HR 1.05, 95%CI:1.02,1.08), azithromycin treatment (HR 5.80, 95%CI:1.29, 25.97). Lower in-hospital mortality was associated with steroid treatment (HR 0.39, 95%CI:0.16, 0.96). We found that receiving up-titrated heparin was an independent factor associated with lower in-hospital mortality. (HR 0.31, 95%CI:0.13, 0.75). Schoenfeld residuals were tested in the final model, and the results showed that the proportional hazards assumption was valid, allowing us to interpret the independent effect of the up-titrated group separate from the effect of steroids.

### Hospitalization and ICU stay

The median hospitalization time was 5 days among all patients, ranging from 1 to 80 days. Those who underwent anticoagulant adjustment experienced more severe clinical conditions. Most of them required intensive care unit (ICU) admission (86.1%) and the use of mechanical ventilation (MV) (66.7%). In contrast, the group with fixed anticoagulant treatment had less severe clinical conditions and a shorter hospitalization duration, with a median stay of 4 days, compared to 21 days in the anticoagulant adjustment group.

The median duration of ICU stay was 7 days for all patients. Patients who received anticoagulant adjustments had significantly longer ICU stays and mechanical ventilation durations when compared to those on fixed anticoagulant treatment. Specifically, in the fixed anticoagulant group, the median ICU stay was 3 days, whereas in the anticoagulant adjustment group extended to 18 days. (p<0.001) Similarly, the median duration of mechanical ventilation use was 10 days in the fixed anticoagulant group, compared to 19 days in the anticoagulant adjustment group. (p<0.001) However, among the patients who died in the hospital. The patients who received anticoagulant adjustment had a longer survival duration, with a median of 22.5 days (ranging from 11 to 68 days), compared to 8.5 days (ranging from 1 to 27 days) in the fixed anticoagulant group.

### Efficacy and safety outcome

Across the cohort, there were 13 patients who experienced bleeding events (5%). Only one major bleeding event (0.38%) occurred in up-titrate anticoagulant group, while 12 patients (4.62%) experienced clinically relevant non-major bleeding (CRNMB) event by ISTH criteria. The prevalence of all type of bleedings were significantly higher in patients who received anticoagulant adjustment compared to who received fixed anticoagulant dosage (12.5% vs 2.13%, p<0.01). There were 7 patients (2.7%) who experienced breakthrough thrombosis. Six patients in the anticoagulant adjustment group developed venous thromboembolism (VTE), while one of the patients in fixed anticoagulant group developed arterial thrombosis. The only arterial thrombotic event that developed in fixed anticoagulant group is an ischemic stroke, and common thrombotic event in anticoagulant adjustment group was deep vein thrombosis (DVT).

***D-dimer analysis***

**Figure 3.**
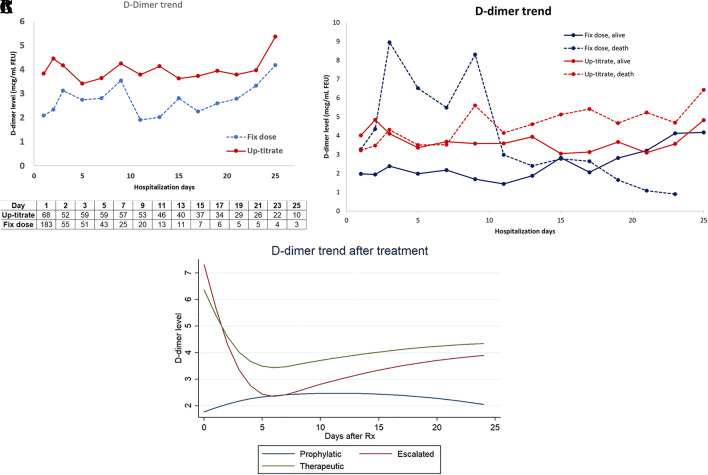
A. Mean profiles of D-dimer over hospitalization days. B. Mean profiles over time of D-dimer according to survival outcome and anticoagulant group. C. Mean profiles over time of D-dimer for each escalated step by random-intercept linear mixed model.

Mean profiles over time of D-dimer levels during hospitalization in both groups are shown in Figure 3A. When comparing the mean D-dimer level trends between patients who survived and those who died, we found a higher trend among the deceased, particularly during the first week of hospitalization for patients who received a fixed-dose anticoagulant as shown in Figure 3B. The suppressible level of D-dimer might explain the survival advantage of the up-titrated group in the early course of the disease. However, the timing of D-dimer level progression varied among patients. The progression of D-dimer levels in patients who died while receiving an up-titrated anticoagulant occurred after the first week. Considering the varying timing of D-dimer progression among patients, we also compared and graphically presented the mean profiles over time of D-dimer levels following each escalation step using a random-intercept linear mixed model. This model accounted for the correlation of D-dimer measurements within individuals. The fixed effects included time measuring D-dimer, treatment groups, and the interaction of these variables. The peak effect of escalated and therapeutic heparin on D-dimer levels clearly occurred within the first week of each escalation treatment as shown in Figure 3C. However, a peak D-dimer level above or equal to 6 mcg/ml FEU (3.0 ug/mL) or below 6 FEU/ml (3.0 ug/mL) was not associated with survival outcome nor was it a confounder; therefore, it was not included in the survival model.

## Discussion

Many reports and observational studies early in the COVID pandemic era suggest a survival benefit for use of anticoagulation among severely ill COVID patients. ^2^^–^^4^ Considering this, we assessed a quality improvement initiative aimed at implementing a D-dimer-guided anticoagulation strategy for COVID-infected inpatients. We chose a D-dimer-based strategy due to the limited availability of radiologic studies during the COVID-19 pandemic. The most recent guidelines support the use of regular thromboprophylaxis for severely ill COVID-19 patients, as starting with therapeutic-dose heparin as an initial approach did not improve the chances of survival until hospital discharge or having more days without requiring cardiovascular or respiratory organ support. ^12^ On the other hand, the use of therapeutic-dose heparin in non-severely ill COVID-19 patients reduces the need for mechanical ventilation and improves overall survival. ^6^ Our strategy was dynamically adjusted upon the patient’s condition and d-dimer. We stratified patients into three groups, based on D-dimer level on admission and followed a stepwise up-titration of anticoagulation based on D-dimer values, which could occur at any time during the hospitalization. We found that there was no survival benefit for 6 patients who received a fixed therapeutic dose from the beginning of their hospitalization when compared to those who were up-titrated later in their hospital course. This suggests that the dynamic adjustment of anticoagulation treatment yields better survival outcomes compared to a fixed anticoagulant dose, whether it was fixed prophylactic or fixed therapeutic anticoagulation. We observed a suppression of D-dimer values within the first week of using higher doses of anticoagulation, suggesting that up-titrating anticoagulation may mitigate the expected adverse impact on survival. When defining COVID-19 severity according to standard guidelines, the severity is classified as mild for those with mild symptoms. Moderate disease is defined by the presence of clinical or radiographic evidence of lower respiratory tract disease but with a blood oxygen saturation of 94% or higher, and it requires hospitalization. Indicators of severe disease include hypoxemia with oxygen saturation ≤93% or a ratio of partial pressure of arterial oxygen to fraction of inspired oxygen <300. Critical illness is defined by respiratory failure requiring mechanical ventilation or shock requiring vasopressors. According to this definition, we found that 40.9% of patients in the fixed-dose group had severe disease, compared to 15.3% in the up-titrate group. Additionally, there were more critical patients in the up-titrate group, with 72.2% compared to 18.1% in the fixed-dose population. After reclassifying the patients according to standard guidelines, the combination of severe and critical patients accounted for the majority in both groups. The multivariable Cox regression analysis, including severity classified by standard guidelines, shows that severity is an independent factor affecting survival outcomes, while azithromycin treatment has no effect on survival. Therefore, the effect of azithromycin on survival in our data was confounded by patient severity.

An insuppressible trend of D-dimer level among the deceased could explain the worse outcomes in fix dose group. It is possible that deceased patients in the fixed-dose group might have had untreated asymptomatic pulmonary embolisms. According to previous reports, the incidence of VTE among critically ill patients is around 50%, ^4^^,^^5^ compared to 2.7% in our study, where one-third of the population and 84.8% of decrease patients could be classified as critically ill according to standard guidelines. The overall incidence of breakthrough thrombosis in our study is notably low. Furthermore, the mean D-dimer levels over time in deceased patients treated with fixed-dose thromboprophylaxis ( Figure 3B) seemed to fall within the D-dimer range associated with pulmonary embolism (2.0-9.6 mcg/ml FEU) from a systematic review. ^9^ These patients might require early titration of anticoagulants, additional treatment options, or a combination of both.

The prevalence of bleeding events was higher in the patients who received up-titrate doses of anticoagulation. The one major bleeding event observed was due to esophageal varices in a patient with previously undiagnosed liver cirrhosis, while others are clinically relevant non-major bleeding (CRNMB) that managed with standard supportive care and bleeding was not independently associated with survival outcome. The overall incidence of breakthrough thrombotic events was 2.7%, but this is likely underestimated due to a limited number of diagnostic studies performed in era of new emerging COVID disease.

The strengths of our D-dimer-titrated anticoagulation strategy included the use of a simple diagnostic test to stratify patient risk and allow for a dynamic reassessment over the course of the hospitalization. Using the rise in D- dimer as a trigger to increase anticoagulant allowed us to intervene early in the disease course. Our study is limited by its retrospective nature and lack of uniform diagnostic evaluation for DVT/PE in COVID-infected patients, even in those with suggestive symptoms. Previously, Demelo-Rodriguez et al reported that 15% of patients with COVID and elevated D-dimer levels have asymptomatic DVT. ^13^ COVID infection is notably associated with a high risk of thrombotic complications, even in anticoagulated patients; therefore, the number of VTE reported in our cohort is likely underestimated. Furthermore, we did not collect data regarding thrombotic events that might have occurred after patients were discharged, and no autopsies were performed at our site to confirm the causes of death. Finally, we did not include a de-escalation strategy in our guideline, so this was done inconsistently based on physician discretion.

In this quality improvement evaluation of a D-dimer-titrated anticoagulation strategy for hospitalized patients with COVID, we found that D-dimer combined with clinically-driven anticoagulation adjustment was associated with reduced in-hospital mortality with a manageable increase in minor bleeding events.

## Consent to participate

Informed consent waiver was obtained by University of Southern California Institutional Review Board. Based on the U.S. Code of Federal Regulations (CFR), the IRB determined that this study was exempt from 45 CFR 46 according to §46.104(d) as category 4 – Exempt Research as follows:

“(4) Secondary research for which consent is not required: Secondary research uses of identifiable private information or identifiable biospecimens, if at least one of the following criteria is met:

(i) The identifiable private information or identifiable biospecimens are publicly available;

(ii) Information, which may include information about biospecimens, is recorded by the investigator in such a manner that the identity of the human subjects cannot readily be ascertained directly or through identifiers linked to the subjects, the investigator does not contact the subjects, and the investigator will not re-identify subjects;

(iii) The research involves only information collection and analysis involving the investigator’s use of identifiable health information when that use is regulated under 45 CFR parts 160 and 164, subparts A and E, for the purposes of “health care operations” or “research” as those terms are defined at 45CFR 164.501 or for “public health activities and purposes” as described under 45 CFR 164.512(b);

(iv) The research is conducted by, or on behalf of, a Federal department or agency using government-generated or government-collected information obtained for non research activities, if the research generates identifiable private information that is or will be maintained on information technology that is subject to and in compliance with section 208(b) of the E-Government Act of2002, 44 U.S.C. 3501 note, if all of the identifiable private information collected, used, or generated as part of the activity will be maintained in systems of records subject to the Privacy Act of 1974, 5U.S.C. 552a, and, if applicable, the information used in the research was collected subject to the Paperwork Reduction Act of 1995, 44 U.S.C. 3501 et seq.”

Ethical approval: Based on the review conducted by the University of Southern California Institutional Review Board (IRB) designee on April 13, 2021, the study has been evaluated and categorized as exempt. The study is authorized to proceed with conducting as approved by the IRB. This exemption indicates that the research poses minimal risk to participants or involves only the collection of existing data, among other criteria outlined in the regulations.

## Author contribution statement

1. Lantarima Bhoopat performed study design, investigation, data curation, formal analysis, data interpretation, writing - original draft, writing - review & editing.
2. Anastasia Martynova performed investigation, data curation, data interpretation, writing- original draft, writing - review & editing.
3. April Choi performed investigation, data curation, writing – original draft, writing -review & editing.
4. Pattharawin Pattharanitima performed formal analysis, review & editing.
5. Semi Han performed Data acquisition, review & editing.
6. Senxi Du performed data abstraction, review & editing.
7. Ibrahim Syed performed data curation, review & editing.
8. Catherine Chan performed data curation, review & editing.
9. Esther E Oh performed data curation, investigation, review & editing.
10. Zea Borok performed conceptualization, review and editing.
11. Janice Liebler performed conceptualization, review and editing.
12. Melissa Lee Wilson performed data analysis and interpretation, drafting statistical methods section, writing- review and editing.
13. Pichaya Tantiyavarong performed data analysis, data interpretation, writing-review & editing.
14. Casey O’Connell performed conceptualization, project administration, supervision, validation, writing – review & editing.

## Data Availability

Zenodo: A Dynamic, D-dimer-based Thromboprophylaxis Strategy in Patients with COVID-19, https://doi.org/10.5281/zenodo.12687462. ^14^ The project contains the following underlying data covidheparin_deidentified.xls. Data are available under the terms of the Creative Commons Attribution 4.0 International license (CC-BY 4.0) Zenodo: STROBE checklist for A Dynamic, D-dimer-based Thromboprophylaxis Strategy in Patients with COVID-19. Zenodo. https://doi.org/10.5281/zenodo.12730540. ^15^ The project contains the following underlying data STROBE_checklist-ddCOVID.pdf Data are available under the terms of the Creative Commons Attribution 4.0 International license (CC-BY 4.0). Dataset available at https://doi.org/10.5281/zenodo.10649845
