# Supplementary material for: A Dynamic, D-dimer-based Thromboprophylaxis Strategy in Patients with COVID-19
Source: F1000Res. 2024 Nov 11;13:887. Originally published 2024 Aug 5. [Version 3] doi: 10.12688/f1000research.146710.3 (PMC11467651; doi:10.12688/f1000research.146710.3)
Supplement: Peer review history

# Reviewer report: Reviewer response for version 2

**DOI:** 10.5256/f1000research.171179.r324949

**Published:** 2024-10-16

**Authors:** Bundarika Suwanawiboon

**Affiliations:** 1 Mahidol University, Salaya, Nakhon Pathom, Thailand

Bhoopat and co-authors reported the efficacy and safety of a dynamic thromboprophylaxis strategy in a cohort of hospitalized patients with COVID-19 infection who received fixed or up-titrated doses of anticoagulation. Their anticoagulant protocol allowed for the adjustment of thromboprophylaxis doses based on an increase in D-dimer levels, the inability to undergo dialysis due to clotting, or acute respiratory decompensation. This approach is practical during the pandemic when access to radiographic studies for early detection of venous thromboembolism (VTE) is limited. Furthermore, the study provided informative data that could help enhance thromboprophylaxis protocols for hospitalized COVID-19 patients.

 However, several issues require further clarification.

 1. The authors acknowledged the study's limitations in determining causes of death through autopsies. However, could the authors provide information on the most common causes of death, if possible?

 Were there any differences in thromboembolism-related mortality between the two treatment groups?

 2. In addition to the fixed doses of anticoagulation and insuppressible trend of D-dimer levels in deceased patients, as shown in Figure 3B, were there any differences between deceased patients in the fixed-dose group and those with the up-titrating group regarding factors that might affect survival outcomes, for example, age, the clinical severity of COVID-19 according to the standard classification, and COVID-19 treatment?

 3. The authors addressed the limitations of the study, such as the inconsistent diagnostic evaluation of VTE in COVID-19 patients and the absence of autopsies to confirm the causes of death. Furthermore, the mean D-dimer levels over time in deceased patients treated with fixed-dose thromboprophylaxis (Figure 3B) seemed to fall within the D-dimer range associated with pulmonary embolism (2.0-9.6 mcg/ml FEU), as mentioned in the introduction.

 Would the authors consider the possibility that deceased patients in the fixed-dose group might have had untreated asymptomatic pulmonary embolisms, which might result in lower in-hospital survival compared with those in the up-titrating group?

 4. If the data was available, was there any correlation between the suppressible trend of D-dimer levels and the levels of other inflammatory markers, for example, LDH and CRP?

Is the work clearly and accurately presented and does it cite the current literature?

Yes

If applicable, is the statistical analysis and its interpretation appropriate?

Yes

Are all the source data underlying the results available to ensure full reproducibility?

Yes

Is the study design appropriate and is the work technically sound?

Yes

Are the conclusions drawn adequately supported by the results?

Yes

Are sufficient details of methods and analysis provided to allow replication by others?

Yes

Reviewer Expertise:

Thrombosis and hemostasis

I confirm that I have read this submission and believe that I have an appropriate level of expertise to confirm that it is of an acceptable scientific standard.

# Response

**Published:** 2024-11-03

**Authors:** Lantarima Bhoopat

I would like to thank you the reviewer for the review and comments. I would address the queries as below.

 1. The authors acknowledged the study's limitations in determining causes of death through autopsies. However, could the authors provide information on the most common causes of death, if possible?

 Were there any differences in thromboembolism-related mortality between the two treatment groups?

- *All causes of death are determined to be due to COVID infection. We apologize for not collecting specific data on thromboembolism-related mortality. However, there were 2 non-surviving patients who were confirmed breakthrough thrombosis: 1 with deep vein thrombosis (DVT) and another with pulmonary embolism along with DVT.*

2. In addition to the fixed doses of anticoagulation and insuppressible trend of D-dimer levels in deceased patients, as shown in Figure 3B, were there any differences between deceased patients in the fixed-dose group and those with the up-titrating group regarding factors that might affect survival outcomes, for example, age, the clinical severity of COVID-19 according to the standard classification, and COVID-19 treatment?

- *When defining COVID-19 severity according to standard guidelines, the severity is classified as mild for those with mild symptoms. Moderate disease is defined by the presence of clinical or radiographic evidence of lower respiratory tract disease but with a blood oxygen saturation of 94% or higher, and it requires hospitalization. Indicators of severe disease include hypoxemia with oxygen saturation ≤93% or a ratio of partial pressure of arterial oxygen to fraction of inspired oxygen <300. Critical illness is defined by respiratory failure requiring mechanical ventilation or shock requiring vasopressors.*
- *Among non-surviving patients, we found that 31.2% of patients in the fixed-dose group were classified as severe and 68.2% as critical, while all of the non-surviving patients in the up-titrated group were classified as critical illness.*
- *After reclassifying the patients according to standard guidelines and including the severity classification as a factor in the multivariate analysis of the survival model, we found that critical illness became an independent factor affecting the outcome, and azithromycin treatment had no effect on survival. Therefore, the effect of azithromycin on survival was confounded by patient severity.*
- *Other factors affecting survival that showed statistically significant differences between non-surviving patients in both groups included age and steroid treatment. The fixed-dose group had a higher median age of 82 years (range 37-97), while the up-titrated group had a median age of 59 years (range 30-76). However, non-surviving patients in the up-titrated group received more steroid treatment (70.6%) compared to 25% in the fixed-dose group.*

3. The authors addressed the limitations of the study, such as the inconsistent diagnostic evaluation of VTE in COVID-19 patients and the absence of autopsies to confirm the causes of death. Furthermore, the mean D-dimer levels over time in deceased patients treated with fixed-dose thromboprophylaxis (Figure 3B) seemed to fall within the D-dimer range associated with pulmonary embolism (2.0-9.6 mcg/ml FEU), as mentioned in the introduction.

 Would the authors consider the possibility that deceased patients in the fixed-dose group might have had untreated asymptomatic pulmonary embolisms, which might result in lower in-hospital survival compared with those in the up-titrating group?

- *It is possible that deceased patients in the fixed-dose group might have had untreated asymptomatic pulmonary embolisms. According to previous reports, the incidence of VTE among critically ill patients is around 50%, compared to 2.7% in our study, where most of the population could be classified as critically ill according to standard guidelines. The overall incidence of breakthrough thrombosis in our study is notably low. These patients might have required earlier titration of anticoagulants, and I will include an additional discussion on this in the discussion section.*

4. If the data was available, was there any correlation between the suppressible trend of D-dimer levels and the levels of other inflammatory markers, for example, LDH and CRP?

- *There are some correlations between the suppressible trend of D-dimer levels and the levels of other inflammatory markers, especially CRP, as shown in the figure below.*

# Response

**Published:** 2024-11-03

**Authors:** Lantarima Bhoopat

There are some correlations between the suppressible trend of D-dimer levels and the levels of other inflammatory markers, especially CRP, as shown in the figure below.

*https://f1000research.s3.amazonaws.com/linked/688634.146710-Figure_files.pdf*

# Reviewer report: Reviewer response for version 2

**DOI:** 10.5256/f1000research.171179.r324950

**Published:** 2024-10-10

**Authors:** Adisak Tantiworawit

**Affiliations:** 1 Chiang Mai University, Chiang Mai, Chiang Mai, Thailand

This is a retrospective study to compare the survival benefit between fixed and up-titrated anticoagulation. The treatment was guided by D-dimer. The findings of this study are interesting and may guide treatment for COVID-19 patients. However, there are somel concerns and questions that need to be addressed.

 1. Which factors was used to analyze in the  multivariable Cox regression for survival outcome?

 2. Why does azithromycin treatment was the factors associated for survival outcome, please give more discussion and answer?

 3. Please give and discuss more about the limitation of the study such as

 - The imbalance characteristics  between group such as steroid treatment, antiviral treatment

 - The survival outcome may effect by other treatment, not only anticoagulant treatment

Is the work clearly and accurately presented and does it cite the current literature?

Yes

If applicable, is the statistical analysis and its interpretation appropriate?

Partly

Are all the source data underlying the results available to ensure full reproducibility?

Yes

Is the study design appropriate and is the work technically sound?

Yes

Are the conclusions drawn adequately supported by the results?

Yes

Are sufficient details of methods and analysis provided to allow replication by others?

Partly

Reviewer Expertise:

hematology

I confirm that I have read this submission and believe that I have an appropriate level of expertise to confirm that it is of an acceptable scientific standard.

# Response

**Published:** 2024-10-16

**Authors:** Lantarima Bhoopat

I would like to thank you the reviewer for the review and comments. I would address the queries as below.

 1. Which factors was used to analyze in the multivariable Cox regression for survival outcome?

*We evaluated the following potential covariates: D-dimer levels, age, ethnicity, BMI, comorbidities, ICU admission, mechanical ventilation, HFNC, SIC score, COVID treatment, thrombosis, and bleeding events in the multivariable Cox regression for survival outcome.*

 2. Why does azithromycin treatment was the factors associated for survival outcome, please give more discussion and answer?

*We have certain limitations in answering this question. As we did not collect data on the timing of treatment initiation, we are unable to evaluate the clinical condition at the time azithromycin was started, or whether it was administered too late in the course of the disease.*

 3. Please give and discuss more about the limitation of the study such as

 - The imbalance characteristics between group such as steroid treatment, antiviral treatment

*The imbalance in treatment characteristics between the groups could be explained by the difference in severity between them. Patients who received anticoagulant adjustment were treated more aggressively for COVID-19 infection, with therapies such as remdesivir, azithromycin, hydroxychloroquine, convalescent plasma, and steroids, in response to their severe clinical course. The severity of patients in the anticoagulant adjustment group was reflected in higher rates of ICU admission, high-flow nasal cannula (HFNC) requirement, mechanical ventilator (MV) requirement, and vasopressor use compared to the fixed anticoagulant group. The sepsis-induced coagulopathy scores (SICS) were also significantly higher in this group, correlating with higher lactate dehydrogenase (LDH) levels and d-dimer levels both at baseline and at their peak during admission.*

 - The survival outcome may effect by other treatment, not only anticoagulant treatment

*Although steroid treatment was associated with lower in-hospital mortality (HR 0.39, 95% CI: 0.16, 0.96), we demonstrated that receiving up-titrated heparin was an independent factor associated with reduced in-hospital mortality. This was confirmed using Schoenfeld residuals tested in the final model, which showed that the proportional hazards assumption was valid, allowing us to interpret the independent effect of the up-titrated group separately from the effect of steroids.*

# Response

**Published:** 2024-10-20

**Authors:** Lantarima Bhoopat

I re-analyzed the data today and now have more supporting evidence to answer the reviewer's second question regarding why azithromycin treatment was a factor associated with survival outcomes, as outlined below

 Initially, we did not classify the patients according to standard guidelines, which categorize the severity as follows: mild for those with mild symptoms; moderate disease is defined by the presence of clinical or radiographic evidence of lower respiratory tract disease but with a blood oxygen saturation of 94% or higher and requiring hospitalization; severe disease is indicated by hypoxemia with oxygen saturation ≤93% or a ratio of partial pressure of arterial oxygen to fraction of inspired oxygen <300; and critical illness is defined by respiratory failure requiring mechanical ventilation or shock requiring vasopressors.

 After reclassifying the patients according to standard guidelines and including the new severity classification as a factor in the multivariate analysis of the survival model, we found that azithromycin treatment had no effect on survival. However, critical patients became an independent factor affecting the outcome, which may support the assumption that the effect of azithromycin on survival was confounded by patient severity.

# Reviewer report: Reviewer response for version 2

**DOI:** 10.5256/f1000research.171179.r320738

**Published:** 2024-09-18

**Authors:** Prof. NOPPACHARN UAPRASERT

**Affiliations:** 1 Chulalongkorn University, Bangkok, Bangkok, Thailand

The authors have adequately addressed all of my comments.

Is the work clearly and accurately presented and does it cite the current literature?

Yes

If applicable, is the statistical analysis and its interpretation appropriate?

Partly

Are all the source data underlying the results available to ensure full reproducibility?

No source data required

Is the study design appropriate and is the work technically sound?

Yes

Are the conclusions drawn adequately supported by the results?

Yes

Are sufficient details of methods and analysis provided to allow replication by others?

Partly

Reviewer Expertise:

Thrombosis and Hemostasis

I confirm that I have read this submission and believe that I have an appropriate level of expertise to confirm that it is of an acceptable scientific standard.

# Reviewer report: Reviewer response for version 1

**DOI:** 10.5256/f1000research.160824.r311489

**Published:** 2024-08-19

**Authors:** Prof. NOPPACHARN UAPRASERT

**Affiliations:** 1 Chulalongkorn University, Bangkok, Bangkok, Thailand

The authors conducted a retrospective study to compare the survival benefit between fixed and up-titrated anticoagulation guided by D-dimer levels. The findings of this study are very interesting and may guide anticoagulation for hospitalized COVID-19 patients. However, there are several concerns that need to be addressed. 

 1. Patients should be classified as mild, moderate, severe, or critical according to standard guidelines, as mortality is likely to be associated with their severity.

 2. Dynamic changes in D-dimer levels in both groups during hospitalization should be demonstrated in the results section. 

 3. It is apparent that patients receiving up-titrated anticoagulants had greater severity, received more intensive treatment (including steroids), and had a longer ICU stay. How did the authors explain the lower mortality in more severe COVID-19 patients?

 4. Were the D-dimer levels suppressed after escalated doses of anticoagulants? Was the suppression of D-dimer associated with survival? The authors should demonstrate the results if available and address this in the discussion.

 5. Due to the small sample size in the up-titrated group, it is very difficult to make a definitive association in multivariate analysis. For example, the survival benefit of anticoagulation may depend on steroids. Therefore, this limitation needs to be clearly addressed.

Is the work clearly and accurately presented and does it cite the current literature?

Yes

If applicable, is the statistical analysis and its interpretation appropriate?

Partly

Are all the source data underlying the results available to ensure full reproducibility?

No source data required

Is the study design appropriate and is the work technically sound?

Yes

Are the conclusions drawn adequately supported by the results?

Yes

Are sufficient details of methods and analysis provided to allow replication by others?

Partly

Reviewer Expertise:

Thrombosis and Hemostasis

I confirm that I have read this submission and believe that I have an appropriate level of expertise to confirm that it is of an acceptable scientific standard, however I have significant reservations, as outlined above.

# Response

**Published:** 2024-08-28

**Authors:** Lantarima Bhoopat

I would like to thank you the reviewer for the review and comments. I would address the queries as below.

 1. Patients should be classified as mild, moderate, severe, or critical according to standard guidelines, as mortality is likely to be associated with their severity.

**Author Response: **When defining COVID-19 severity according to standard guidelines, the severity is classified as mild for those with mild symptoms. Moderate disease is defined by the presence of clinical or radiographic evidence of lower respiratory tract disease but with a blood oxygen saturation of 94% or higher, and it requires hospitalization. Indicators of severe disease include hypoxemia with oxygen saturation ≤93% or a ratio of partial pressure of arterial oxygen to fraction of inspired oxygen <300. Critical illness is defined by respiratory failure requiring mechanical ventilation or shock requiring vasopressors.

 According to this definition, we found that 50.5% of patients in the fixed-dose group had severe disease, compared to 20.8% in the up-titrate group. Additionally, there were more critical patients in the up-titrate group, with 47.2% compared to 6.4% in the fixed-dose population.

 After reclassifying the patients according to standard guidelines, the combination of severe and critical patients accounts for more than half of both groups. 

 2. Dynamic changes in D-dimer levels in both groups during hospitalization should be demonstrated in the results section. 

**Author Response: **We added D-dimer analysis section as reviewer suggests. Mean profiles over time of D-dimer levels during hospitalization in both groups are shown in Figure 3A. The timing of D-dimer level progression varied among patients. Thus, we also compared and graphically presented the mean profiles over time of D-dimer after each escalation step using a random-intercept linear mixed model, as shown in Figure 3C. The peak effect of escalated and therapeutic heparin on D-dimer levels clearly occurred within the first week of each escalation step and might affect outcomes in the up-titrate group that will address in the 4 ^th^ question.

*Please refer to Figure 3A here - https://f1000research.s3.amazonaws.com/linked/672277.146710_Figure_3A.pdf*

 Figure 3A: Mean profiles of D-dimer over hospitalization days

 3. It is apparent that patients receiving up-titrated anticoagulants had greater severity, received more intensive treatment (including steroids), and had a longer ICU stay. How did the authors explain the lower mortality in more severe COVID-19 patients?

**Author Response: **After reclassifying the patients according to standard guidelines, the combination of severe and critical patients accounts for more than half of both groups. The d-dimer analysis shows an insuppressible trend among the deceased, which might explain the worse outcomes in this population. These patients might require early titration of anticoagulants, additional treatment options, or a combination of both. We will add on this information in the discussion section.

 4. Were the D-dimer levels suppressed after escalated doses of anticoagulants? Was the suppression of D-dimer associated with survival? The authors should demonstrate the results if available and address this in the discussion.

**Author Response: **When comparing the mean D-dimer level trends between patients who survived and those who died, we found a higher trend among the deceased, particularly during the first week of hospitalization for patients who received a fixed-dose anticoagulant. The progression of D-dimer levels in patients who died while receiving an up-titrated anticoagulant occurred after the first week, as shown in Figure 3B. The suppressible level of D-dimer might explain the survival advantage of the up-titrated group in the early course of the disease. Considering the different timing of D-dimer progression in each patient, we also compared and graphically presented the mean profiles over time of D-dimer levels after each escalation step using a random-intercept linear mixed model, as shown in Figure 3C. This model accounted for the correlation of D-dimer measurements within individuals. The fixed effects included time measuring D-dimer, treatment groups, and the interaction of these variables. A restricted cubic spline with 3 degrees of freedom was applied to relax the linearity assumption. The peak effect of escalated and therapeutic heparin on D-dimer levels clearly occurred within the first week of each escalation treatment (as shown in Figure 3C). However, a peak D-dimer level above or equal to 6 mcg/ml FEU (3.0 ug/mL) or below 6 FEU/ml (3.0 ug/mL) was not associated with survival outcome nor was it a confounder; therefore, it was not included in the survival model.

*Please refer to Figure 3B here - https://f1000research.s3.amazonaws.com/linked/672278.146710_Figure_3B.pdf*

 Figure 3B: Mean profiles over time of D-dimer according to survival outcome and anticoagulant group.

*Please refer to Figure 3C here - https://f1000research.s3.amazonaws.com/linked/672279.146710_Figure_3C.pdf*

 Figure 3C: Mean profiles over time of D-dimer for each escalated step by random-intercept linear mixed model

 5. Due to the small sample size in the up-titrated group, it is very difficult to make a definitive association in multivariate analysis. For example, the survival benefit of anticoagulation may depend on steroids. Therefore, this limitation needs to be clearly addressed.

**Author Response: **Upon the reviewer suggested, we added information in survival outcome section to address that limitation as in the Cox proportional hazards model, Schoenfeld residuals were tested in the final model, the results showed that the proportional hazards assumption was valid, allowing us to interpret the independent effect of the up-titrated group separate from the effect of steroids.

# Response

**Published:** 2024-10-20

**Authors:** Lantarima Bhoopat

I re-analyzed the data today and found some mistakes in the numbers after reclassification according to the standard guidelines that I addressed in my response to the reviewer's first question in paragraph 2. I would like to correct them as follows.

 According to this definition, we found that 40.9 % of patients in the fixed-dose group had severe disease, compared to 15.3% in the up-titrate group. Additionally, there were more critical patients in the up-titrate group, with 72.2% compared to 18.1% in the fixed-dose population. After reclassifying the patients according to standard guidelines, the combination of severe and critical patients accounts for more than half of both groups.
